# Claudin-18 expression in small bowel adenocarcinoma: a clinico-pathologic study

**DOI:** 10.1007/s00428-022-03393-6

**Published:** 2022-08-04

**Authors:** Giovanni Arpa, Matteo Fassan, Camilla Guerini, Erica Quaquarini, Federica Grillo, Valentina Angerilli, Vincenza Guzzardo, Sara Lonardi, Francesca Bergamo, Marco Vincenzo Lenti, Paolo Pedrazzoli, Marco Paulli, Antonio Di Sabatino, Alessandro Vanoli

**Affiliations:** 1 Department of Molecular Medicine, Unit of Anatomic Pathology, University of Pavia, Via Carlo Forlanini 16-27100, Pavia, Italy; 2 Anatomic Pathology Unit, Fondazione IRCCS San Matteo Hospital, Pavia, Italy; 3 Department of Medicine, DIMED, University of Padua, Padua, Italy; 4 Veneto Institute of Oncology, IOV-IRCCS, Padua, Italy; 5 Medical Oncology Unit, ICS Maugeri-IRCCS SpA SB, 27100 Pavia, Italy; 6 Pathology Unit, Department of Surgical and Diagnostic Sciences (DISC), University of Genoa, Genoa, Italy; 7 IRCCS Ospedale Policlinico San Martino, Genoa, Italy; 8 Department of Oncology, Veneto Institute of Oncology, IOV-IRCCS, Padua, Italy; 9 First Department of Internal Medicine, San Matteo Hospital Foundation, University of Pavia, Pavia, Italy; 10 Oncology Unit, IRCCS San Matteo Hospital, Pavia, Italy

**Keywords:** Coeliac disease, Crohn’s disease, Gastric markers, Immune-mediated disorder, Small intestine

## Abstract

Non-ampullary small bowel adenocarcinoma is a rare neoplasm with an ominous prognosis, whose incidence is higher in some chronic immuno-inflammatory conditions, such as coeliac and Crohn’s disease. Recently, claudin 18.2, a transmembrane protein normally expressed in gastric mucosa, has been recognized as a novel pan-cancer therapeutic target, and several clinical trials with claudin-18-directed drugs have shown promising results on various gastrointestinal malignancies. This is the first study focusing on claudin-18 expression in small bowel adenocarcinomas. The immunohistochemical expression of claudin-18 (clone 43-14A) was assessed in 81 small bowel adenocarcinomas of diverse aetiologies and correlated with several clinico-pathologic features and patient survival. We found that 28% of adenocarcinomas were immunoreactive for claudin-18, with cutoff values of ≥1% at any intensity, while 6% of cancers showed immunoexpression of ≥75% with 2+/3+ score. Moreover, claudin-18 (≥1%) was positively associated with cytokeratin 7 (CK7) and MUC5AC expression, showing CK7+/MUC5AC+ carcinomas the highest rate of positive cases, whereas a negative correlation was found between claudin-18 and CDX2 expression. In addition, some cancer-adjacent dysplastic growths and foci of gastric-type metaplasia in Crohn’s disease-associated cases showed claudin-18 immunoreactivity. Survival analysis showed a non-significant trend towards a worse cancer-specific survival for claudin-18-positive cases. A fraction of small bowel adenocarcinomas, mainly sporadic or Crohn’s disease-associated, and often exhibiting a non-intestinal immunoprofile, expressed claudin-18, suggesting that claudin-18-directed targeted therapy is worth investigating in such cancers.

## Introduction

Non-ampullary small bowel adenocarcinoma (SBA) is a rare epithelial neoplasm representing 30–40% of all cancers [1] of the small intestine and featuring an increasing worldwide incidence [2 3] and a dismal prognosis [4]. Besides de novo sporadic SBAs (Spo-SBAs), hereditary tumour syndromes and chronic immuno-inflammatory conditions represent major risk factors in developing SBA [5 6]. In fact, a substantial proportion of SBAs arises in a background of coeliac disease (CoD) or Crohn’s disease (CrD), each one characterized by quite peculiar clinicopathological features [7 8]. As SBA-related symptoms at presentation are commonly mild and non-specific and an extensive portion of the small intestine cannot be explored by routine endoscopy, diagnosis is often greatly delayed and reached at advanced stages [4 9 10].

Claudin multigene family comprises 27 transmembrane proteins, which takes part in tight junction strands in epithelial cells, playing a pivotal role in tissue homeostasis (i.e. regulating paracellular ion flux and transport and participating in the maintenance of the luminal barrier) and in recruiting signalling proteins [11 12 13]. Particularly, claudin-18 (CLDN18), which can be found in two splicing variants, has a specific topographic expression in healthy tissues, with claudin-18.1 being expressed in the lung and claudin-18.2 in the stomach [14]. In addition, claudin-18.2 was also identified in several epithelial neoplasms, including gastric, pancreatobiliary, oesophageal, colorectal, ovarian and non-small cell lung carcinomas [15 16 17 18]. However, to the best of our knowledge, no study has hitherto investigated CLDN18 expression in SBAs.

Being selectively expressed only in short-lived differentiated gastric cells, other than in some neoplastic tissues, claudin-18.2 was recognized as a safe pan-cancer target [16 19]. Therefore, both monoclonal antibodies (mAb) [20 21 22] and claudin-18.2-specific chimeric antigen receptor engineered T-cells (CAR-T) [23 24] have been recently developed and employed in several clinical trials, showing promising results in the treatment of advanced gastroesophageal and pancreatobiliary tract cancers. Moreover, immunohistochemistry studies on CLDN18 in cancers have demonstrated a certain linkage between its expression and certain histotypes in both gastric [16 25 26] and non-gastric neoplasms [16 27 28], as well as an inverse association with cancer-specific survival and cancer aggressiveness [25 27 29 30]. Interestingly, claudin-18.2 expression was even identified in gastric metaplasia in the background of Barrett’s oesophagus, an early well-recognized event in the development of oesophageal and oesophago-gastric junction adenocarcinoma [31] as well as in pancreatic intraepithelial neoplasia and other precursor lesions of pancreatic ductal adenocarcinoma [32]. Claudin-18.2 ectopic expression therefore appeared to be associated with the activation of specialized genetic programs and with a sort of lineage commitment towards gastric differentiation in several carcinogenic processes [16]. With regard to this, our group has recently described a significant fraction of CrD-SBAs expressing two further non-intestinal/gastric, *metaplastic* markers, i.e. cytokeratin 7 (CK7) and MUC5AC, both in their invasive components and in the associated non-conventional or atypical lesions, when compared to a control group composed with Spo-SBAs, with resultant, important prognostic implications [33].

The aim of this study was to analyse CLDN18 expression in a rather large series of primary non-ampullary SBAs of different aetiologies and to correlate it with several clinicopathological features and with patient survival.

## Materials and methods

Patients with surgically resected, non-hereditary, non-ampullary SBAs collected through the Small Bowel Cancer Italian Consortium, with available, unstained tumour sections were enrolled in this study. The aetiological association with any immuno-inflammatory disorder was ascertained by serology, imaging, clinical and histologic findings. We excluded ampullary adenocarcinomas from the present study, as they have been found to have distinctive features in comparison with non-ampullary duodenal adenocarcinomas [34]. This study was approved by the Ethics Committee of Pavia (protocol number: 20140003980).

Tissue samples were fixed in 4% formaldehyde and processed in paraffin wax. Four-micrometre-thick sections stained with haematoxylin–eosin of all cases were reinvestigated for the following histologic variables: pTNM stage (according to the 8^th^ edition American Joint Committee on Cancer (AJCC) staging system criteria) [35], histologic grade, lymphovascular and perineural invasion. SBAs were histologically subclassified in cohesive (including the glandular-type and the medullary-type) and non-cohesive histotypes (including the poorly cohesive cell-type and the mixed-glandular-poorly cohesive type) [8 36]. The presence of conventional (flat or raised dysplasia) and/or non-conventional precancerous lesions [37] associated with SBAs was recorded.

For immunohistochemistry (IHC), tissue samples were stained using the following antibodies: CLDN18, (clone 43-14A; Roche Ventana), CK7 (clone OV-TL 12/30, Dako), CDX2 (clone DAK-CDX2, Dako), CK20 (clone Ks20.8, Dako), MUC5AC (clone CLH2, Abcam), MUC6 (clone CLH5; Leica Biosystems Newcastle Ltd, Newcastle Upon Tyne, UK), β-catenin (clone 14/Beta-Catenin, BD).

CLDN18 immunoreactivity was evaluated with a quantitative (percentage of stained tumour cells) and semiquantitative method, using a histoscore (H-score), as reported in previous studies [18 26 38]. For H-score assessment, 3+ score was given if tumour cells showed a strong, membranous, circumferential staining; 2+ score if tumour cells had a membranous strong but incomplete staining or a complete, circumferentially faint staining; 1+ score when tumour cells showed a faint, incomplete membranous immunostaining; 0 score when no membranous immunoreactivity was found in neoplastic cells. Thus, tumour cells expressing different intensity scores (3+, 2+, 1+, 0) were evaluated separately in percentage and added up to a total of 100%. H-score was calculated by the formula: H-score = [0 × percentage of negative tumour cells] + [1 × percentage 1+-scored tumour cells] + [2 × percentage of 2+-scored tumour cells] + [3 × percentage of 3+-scored tumour cells]. The maximum value of H-score was 300, for tumours expressing 3+-scored immunoreactivity in 100% tumour cells. SBAs showing ≥ 1% of immunoreactive (at least 1+ intensity) neoplastic cells were considered as positive. Cases with a 2+/3+ score CLDN18 intensity in ≥ 75% of tumour cells, which is the IHC cutoff being used for eligibility in ongoing zolbetuximab clinical trials (NCT03504397; NCT03653507; NCT03816163), were recorded separately. All the other markers were considered as positive when at least 10% of the neoplastic cells were stained, as previously reported [7 8 33]. On the basis of the expression of gastric (MUC6 and MUC5AC) and intestinal (CK20 and CDX2) immunophenotypic markers, cases were subclassified into three immunoprofiles defined as: (i) gastric (i.e. tumours showing immunoreactivity for MUC5AC and/or MUC6, in the absence of both CDX2 and CK20 expression), (ii) intestinal (i.e. neoplasms exhibiting reactivity for CK20 and/or CDX2 and no expression of both MUC5AC and MUC6) and (iii) hybrid (i.e. SBAs showing a concomitant immunoreactivity for at least one intestinal and at least one gastric marker) profiles. Cases with nuclear accumulation of β-catenin in at least 10% of neoplastic cells were recorded as positive. Mismatch repair (MMR) proteins status was assessed using the following antibodies: MLH1 (monoclonal, clone ES05, Dako), MSH2 (monoclonal, clone FE11, Dako), MSH6 (monoclonal, clone EP49, Dako) and PMS2 (monoclonal, clone EP51, Dako); immunostaining of MMR proteins in tumour cells was considered as MMR-proficient (MMRp) if unequivocal nuclear expression of all four MMR proteins was retained, or MMR-deficient (MMRd) if complete loss of nuclear expression of one or more MMR proteins was observed, in the presence of an adequate internal positive control (intra-tumour inflammatory and stromal cells and non-neoplastic cells).

### Statistical analysis

The data were described with the mean and standard deviation if continuous and with counts and percentages if categorical; they were compared between groups with the Student *t* test or the Fisher/*χ*^2^ test, respectively. Median follow-up was computed with the reverse Kaplan-Meier method. Follow- up was computed from diagnosis of cancer to death or last available follow-up for censored patients. Hazard ratios (HRs) and 95% confidence intervals (CI) were computed using Cox regression. A two-sided *P* value<0.05 was considered statistically significant.

## Results

### Demographic and clinico-pathologic features of SBA cases

We analysed 81 cases of surgical resected, non-ampullary, non-hereditary SBAs, encompassing 35 Spo-SBAs, 31 CrD-associated SBAs (CrD-SBAs) and 15 CoD-associated SBAs (CoD-SBAs), part of which have already been included in previous studies [7 8 33 39 40 41]. Their demographic and clinico-pathologic data are summarized in Table 1.

**Table 1:** Comparison of clinico-pathologic and immunophenotypic features between claudin 18-positive and claudin 18-negative non-ampullary small bowel adenocarcinomas

		Total	CLDN18 +	CLDN18-	*P* value
Number of cases		81	23	58	
Age at SBA diagnosis, years mean±SD		62.72±15.52	67.26±14.93	60.91±15.51	0.052
Patient gender, *N* (%)	Female	28 (35)	5 (22)	23 (40)	0.126
	Male	53 (65)	18 (78)	35(60)	
Site, *N* (%)	Duodenum	10 (12)	3(13)	7 (12)	0.059
	Jejunum	30 (37)	4 (17)	26 (45)	
	Ileum	41 (51)	16 (70)	25 (43)	
Aetiology, *N* (%)	Crohn’s disease	31 (38)	12 (52)	19 (33)	0.078
	Coeliac disease	15 (19)	1 (4)	14 (24)	
	Sporadic	35 (43)	10 (44)	25 (43)	
pT stage, *N* (%)	pT1–pT2	7 (9)	5 (22)	2 (3)	**0.018**
	pT3–pT4	74 (91)	18 (78)	56 (97)	
Lymph node metastasis, *N* (%)	Yes	34 (42)	10 (43)	24 (41)	0.836
	No	47 (58)	13 (57)	34 (59)	
Distant metastasis, *N* (%)	Yes	8 (10)	4 (17)	4 (7)	0.153
	No	73 (90)	19 (83)	54 (93)	
AJCC TNM stage, *N* (%)	I–II	47 (58)	13 (57)	34 (59)	1.000
	III–IV	34 (42)	10 (43)	24 (41)	
Lymphovascular invasion, *N* (%)	Yes	54 (67)	17 (74)	37 (64)	0.384
	No	27 (33)	6 (26)	21 (36)	
Perineural invasion, *N* (%)	Yes	30 (37)	10 (43)	20 (34)	0.483
	No	51 (63)	13 (57)	38 (66)	
Histologic grade, *N* (%)	Low grade	54 (67)	18 (78)	36 (62)	0.163
	High grade	27 (33)	5 (22)	22 (38)	
Histotype, *N* (%)	Cohesive	58 (72)	17 (74)	41 (71)	0.772
	Non-cohesive	23 (28)	6 (26)	17 (29)	
CK7 expression, *N* (%)	Yes	33 (41)	19 (83)	14 (24)	**<0.001**
	No	48 (59)	4 (17)	44 (76)	
MUC5AC expression, *N* (%)	Yes	21 (26)	15 (65)	6 (10)	**<0.001**
	No	60 (74)	8 (35)	52 (90)	
CK7/MUC5AC profile, *N* (%)	CK7+/MUC5AC+	18 (22)	14 (61)	4 (7)	<0.001*
	CK7-/MUC5AC-	45 (56)	3 (13)	42 (72)	
	CK7+/MUC5AC-	15 (18)	5 (22)	10 (17)	
	CK7-/MUC5AC+	3 (4)	1 (4)	2 (4)	
MUC6 expression, *N* (%)^	Yes	13 (18)	6 (29)	7 (13)	0.173
	No	61 (82)	15 (71)	46 (87)	
CK20 expression, *N* (%)	Yes	49 (60)	11 (48)	38 (66)	0.169
	No	32 (40)	12 (52)	20 (34)	
CDX2 expression, *N* (%)	Yes	53 (65)	9 (39)	44 (76)	**0.002**
	No	28 (35)	14 (61)	14 (24)	
Immunoprofiles, *N* (%)^	Gastric profile	13 (19)	10 (48)	3 (6)	0.001**
	Intestinal profile	43 (62)	6 (28)	37 (77)	
	Hybrid profile	13 (19)	5 (24)	8 (17)	
β-catenin nuclear expression, *N* (%)^	Yes	28 (39)	3 (18)	25 (45)	**0.049**
	No	44 (61)	14 (82)	30 (55)	
MMRd, *N* (%)	Yes	22 (27)	4 (17)	18 (31)	0.213
	No	59 (73)	19 (83)	40 (69)	

CLDN18-positive SBAs were defined as tumours with at least 1% of CLDN18-positive cells, while the remaining cases were considered CLDN18-negative SBAs

*AJCC* American Joint Committee on Cancer, *CLDN18* claudin 18, *CK* cytokeratin, *MMRd* mismatch repair deficiency, *SBA* small bowel adenocarcinoma, *SD* standard deviation

^*^For post hoc comparisons among CK7/MUC5AC profiles, significance after Bonferroni correction was set at 0.008. Post hoc analysis: CK7+/MUC5AC+ versus CK7−/MUC5AC−, *p*<0.001; the remaining comparisons were not statistically significant (CK7+/MUC5AC+ versus CK7+/MUC5AC−, *p*: 0.015; CK7+/MUC5AC+ versus CK7−/MUC5AC+, *p*: 0.184; CK7−/MUC5AC− versus CK7+/MUC5AC−, *p*: 0.019; CK7−/MUC5AC− versus CK7−/MUC5AC+, *p*: 0.234; CK7+/MUC5AC− versus CK7−/MUC5AC+, p: 1)

^**^For post hoc comparisons among immunoprofiles, significance after Bonferroni correction was set at 0.017. Post hoc analysis: gastric versus intestinal profile: *p*<0.001 with Bonferroni correction; the remaining comparisons were not statistically significant (gastric versus hybrid, *p*: 0.112; intestinal versus hybrid, *p*: 0.104)

^MUC6 and β-catenin were assessed in 74 and 72 cases with available tumour sections, respectively. Immunoprofile could not be assigned in 12 cases because of lack of tumour sections available for MUC6 immunostaining or for absence of expression of both intestinal (CDX2, CK20) and gastric (MUC5AC, MUC6) markers tested

### CLDN18 expression and clinico-pathologic associations in SBAs

**Fig. 1:**
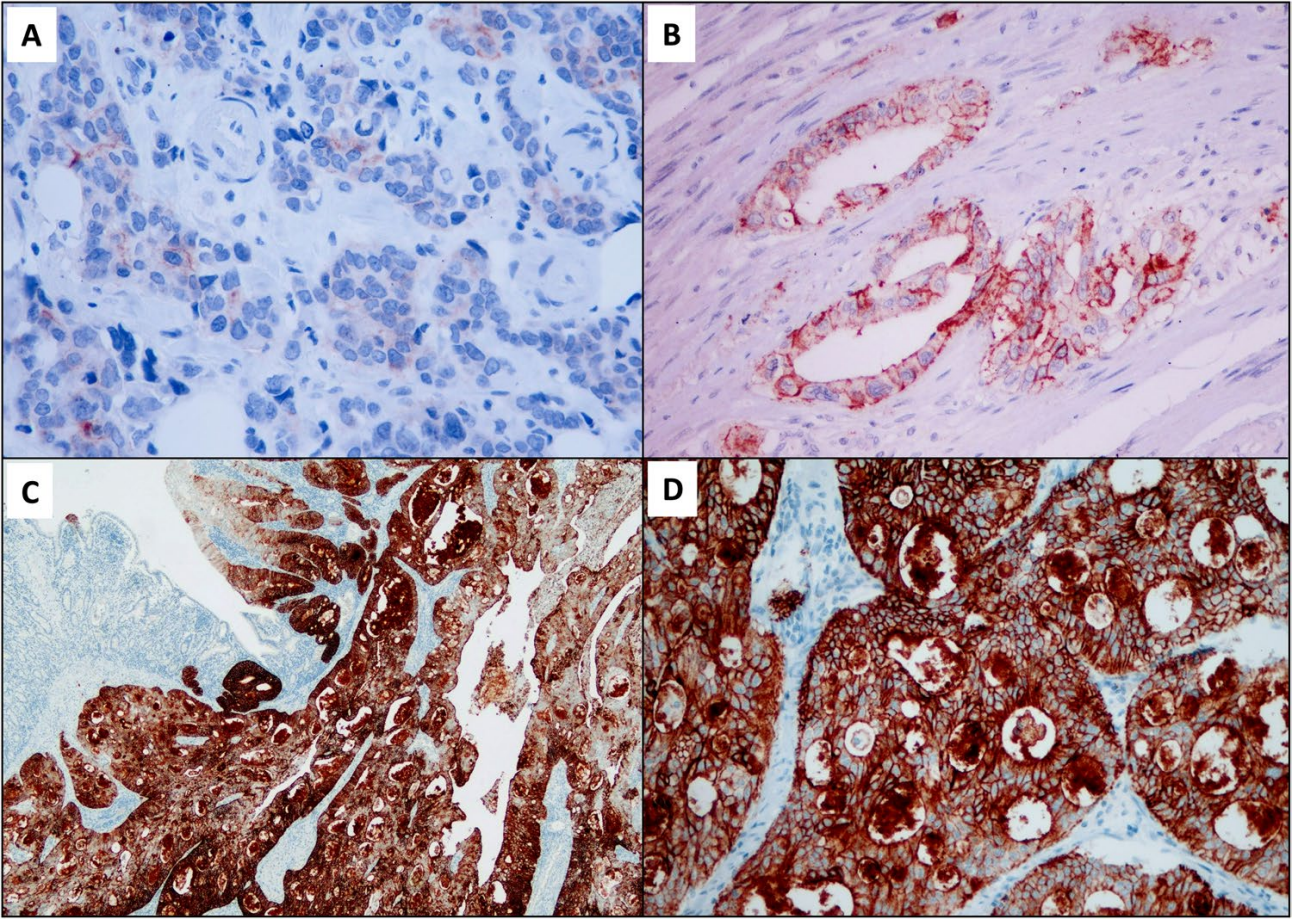
Representative images of claudin-18 immunohistochemical staining in SBAs, showing the spectrum of positive scores: **A,** score 1+; **B,** score 2+; **C–D,** score 3+ (at higher magnification in **D**). **A–D,** Claudin-18 immunohistochemistry; haematoxylin counterstaining

Twenty-three (28%) SBAs showed CLDN18 membranous immunoreactivity in at least 1% of neoplastic cells; the vast majority of these cases had a heterogeneous expression of CLDN18, exhibiting, at different rates, at least two intensity score patterns, with a mean H-score of 68.3 (range 5–270) (Fig. 1).

Clinico-pathologic and immunophenotypic features of the whole cohort and a comparison between CLDN18-positive (≥1% of tumour cells) and CLDN18-negative SBAs (<1%) are summarized in Table 1. A non-significant trend for CLDN18-positive SBAs to occur at older age than CLDN18 negative SBAs was observed (*p*=0.052). Moreover, CLDN18 expression was less frequent in CoD-SBAs (7%) in comparison with CrD-SBAs (39%) and Spo-SBAs (29%) and less common in jejunal tumours (13%) in comparison with duodenal (30%) or ileal (39%) SBAs, although these differences did not reach statistical significance. A statistically significant association between CLDN18 immunoreactivity (≥1% of tumour cells) and lower pT stage was found (*p*=0.018).

**Fig. 2:**
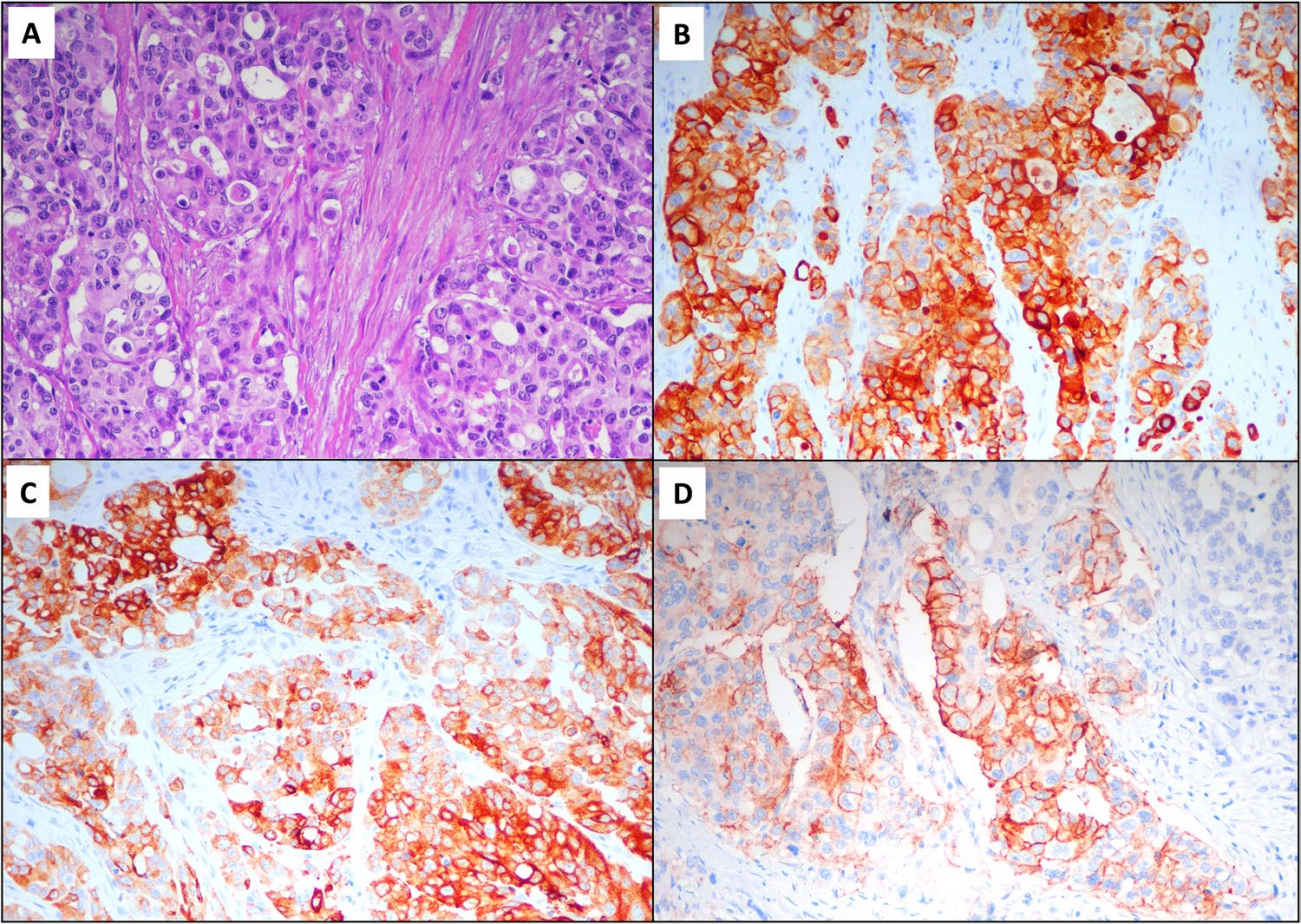
A Crohn’s disease-associated SBA (**A**, haematoxylin and eosin) featuring concomitant expression of cytokeratin 7 (**B**, cytokeratin 7 immunohistochemistry), MUC5AC (**C**, MUC5AC immunohistochemistry) and claudin 18 (**D**, claudin-18 immunostaining)

A strong association was identified between expression of CLDN18 and non-intestinal markers CK7 (*p*<0.001) and MUC5AC (*p*<0.001) (Table 1). On the contrary, CLDN18-positive SBAs expressed CDX2 significantly less frequently (39% versus 76%; *p*=0.002). Stratifying the whole cohort according to the four possible expression patterns for CK7 and MUC5AC, we noted that a great proportion of CLDN18-expressing SBAs (61%) was characterized by a CK7+/MUC5AC+ immunoprofile, while the vast majority of CLDN18-negative SBAs (72%) showed a CK7-/MUC5AC- expression pattern. Interestingly, 14 out of 18 (78%) CK7+/MUC5AC+ SBAs showed immunoreactivity for CLDN18 (Fig. 2). Post hoc comparisons between the four MUC5AC and CK7 expression patterns revealed that CK7+/MUC5AC+ SBAs showed a significantly more common CLDN18 positivity in comparison with CK7-/MUC5AC-SBAs (*p*<0.001), whereas the other comparisons among CK7/MUC5AC patterns did not reach statistical significance, after Bonferroni correction (Table 1). No significant differences were found between CLDN18-positive and CLDN18-negative cases in terms of MUC6 and CK20 expression.

Stratifying our SBA series according to three possible immunophenotypes (intestinal, gastric and hybrid), the only statistically significant difference in the expression of CLDN18 was found between the intestinal and gastric immunoprofile, with SBAs with a gastric profile showing a more frequent expression of CLDN18 (*p*<0.001, after Bonferroni correction, Table 1). Post hoc analysis showed no statistically significant differences between gastric versus hybrid and between intestinal versus hybrid profiles. Finally, a negative association between CLDN18 expression and nuclear β-catenin immunoreactivity was identified (*p*=0.049).

No statistically significant differences between CLDN18-positive and CLDN18-negative SBA cases were found in terms of patient age at diagnosis, patient gender, site, aetiology, AJCC TNM stage, presence of lymph node or distant metastases, lymphovascular or perineural invasion, histologic grade, histotype and MMR status.

Five (6%) SBAs were found to have a moderate-to-strong intensity (i.e. 2+/3+ scores) CLDN18 immunostaining in ≥75% of neoplastic cells (Table 2). Among them, two were located in the ileum, two in the duodenum and one in the jejunum. Of note, all these SBAs arose in male patients, were sporadic, low-grade and with a glandular-type histotype. Most of them (four cases) were MMRp and showed a CK7+/MUC5AC+ immunoprofile. No case with a CLDN18 2+/3+ staining in between 50% and 74% of tumour cells was identified.

**Table 2:** Clinico-pathologic and immunophenotypic features of the five small bowel adenocarcinomas showing claudin 18 immunohistochemical positivity (2+/3+) in ≥75% of tumour cells

Case	Aetiology	Age at SBA diagnosis	Gender	Tumour site	Histological subtype	Grade	pT	AJCC stage	CK7	MUC5AC	CK20	CDX2	MMR-d	Status	Follow-up (mo)
#1	Sporadic	84	M	Duodenum	Glandular	Low	4	4	Pos	Pos	Neg	Neg	No	Alive	9
#2	Sporadic	78	M	Ileum	Glandular	Low	4	2	Pos	Pos	Pos	Neg	Yes	DOD	16
#3	Sporadic	73	M	Ileum	Glandular	Low	3	2	Neg	Neg	Pos	Pos	No	Alive	27
#4	Sporadic	95	M	Jejunum	Glandular	Low	4	2	Pos	Pos	Pos	Pos	No	DOD	5
#5	Sporadic	71	M	Duodenum	Glandular	Low	3	4	Pos	Pos	Neg	Neg	No	DOD	15

*AJCC* American Joint Committee on Cancer, *CK7* cytokeratin 7, *CK20* cytokeratin 20, *DOD* dead of disease, *M* male, *MMR-d* mismatch repair deficiency, *SBA* small bowel adenocarcinoma

**Fig. 3:**
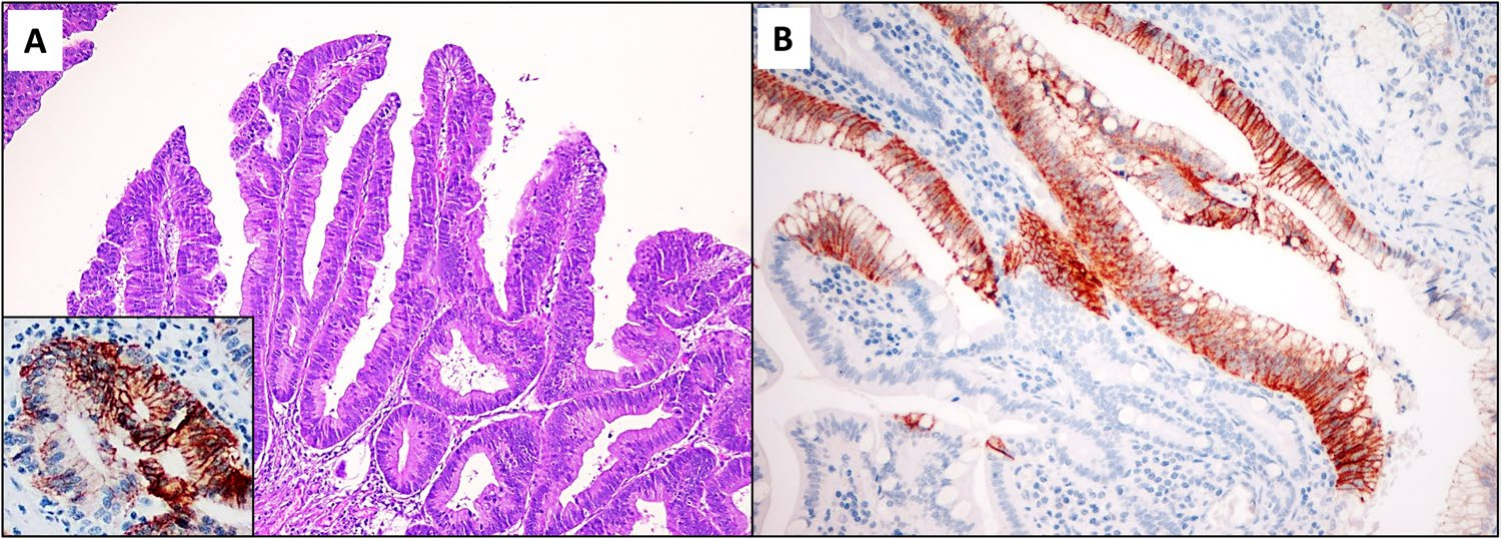
Claudin-18 immunohistochemical expression in precursor/preneoplastic lesions next to SBAs. **A,** A CLDN18-positive conventional adenomatous dysplastic lesion adjacent to a claudin-18-positive sporadic SBA (haematoxylin and eosin; claudin-18 immunostaining in the inlet). **B,** Claudin-18-positive foveolar metaplasia of the surface epithelium adjacent to an SBA associated with Crohn’s disease (claudin-18 immunohistochemistry)

Eighteen cases showed 19 dysplastic lesions adjacent to the SBA, encompassing 11 conventional adenomas, 4 flat-type conventional dysplasia and 4 non-conventional dysplastic lesions; all the non-conventional lesions were detected in association with CrD-SBAs, with two of them observed in the same CrD patient. Five (26%) of such dysplastic growths (two CrD-associated flat conventional dysplasias, two CrD-associated non-conventional lesions and one Spo-SBA-associated conventional adenoma) showed CLDN18-positive cells (Fig. 3A). Interestingly, both CLDN18-positive non-conventional dysplastic growths were associated with the same CLDN18-negative CrD-SBA, while the remaining three CLDN18-positive dysplastic lesions were adjacent to CLDN18-immunoreactive SBAs. Moreover, we noticed that foci of foveolar and pyloric metaplastic epithelium and even some scattered normal-appearing crypts in the mucosa in close proximity of CrD-SBAs expressed CLDN18 (Fig. 3B).

### Survival analysis

Seventy-eight patients were followed up for a median of 41 months, while no follow-up data was available for three cases. Survival analysis showed a trend towards a worse cancer-specific survival for the CLDN18-positive cases in comparison with CLDN18-negative cases, although it did not reach statistical significance (HR: 2.1, 95% CI: 0.98–5.04; *p* value: 0.078).

## Discussion

To date, this is the first study to describe the expression of CLDN18 in a fairly large series of non-ampullary SBAs, including those associated with predisposing inflammatory conditions, and to evaluate its association with several clinico-pathologic features. We found that 28% and 6% of SBAs resulted positive for CLDN18 expression with cutoff values of ≥1% of neoplastic cells at any intensity and ≥75% with 2+/3+ score, respectively. Interestingly, the vast majority of CLDN18-immunoreactive cases were Spo-SBAs or CrD-SBAs, while the prevalence of CLDN18-positivity in CoD-SBAs was low (7%).

Lower rates of CLDN18 positivity were recorded in comparison with those reported in gastroesophageal and bilio-pancreatic cancers [18 20 21 22 25 26 28 42]. Conversely, SBAs expressed CLDN18 more frequently than colorectal carcinomas (CRCs) [27], but, of note, expression rates became similar when CRC cases were enriched, as our series was, with inflammatory bowel disease (IBD)-associated tumours. Moreover, as previously described by Iwaya et al. in the colorectal counterpart [17], we detected CLDN18 expression in tumour-adjacent metaplastic mucosa and, focally, in some scattered, apparently normal crypts of CrD patients only. In our cohort, mostly in CrD-SBAs, CLDN18 positivity was also observed in some cancer-adjacent dysplastic lesions, and in most cases there was concordance in CLDN18 expression between the dysplastic growth and the invasive neoplasm. Our findings are in keeping with prior observations of CLDN18 expression in other gastrointestinal metaplastic and preinvasive lesions (i.e. Barrett’s oesophagus and pancreatic intraepithelial neoplasia) [31 32]. Furthermore, we found a statistically significant association of CLDN18 with both the expression of the non-intestinal markers MUC5AC and CK7 separately evaluated and the MUC5AC+/CK7+ immunophenotype. Interestingly, a correlation of CLDN18 and MUC5AC was previously described in IBD-associated CRCs [17 27]. The increased expression of other metaplastic markers (e.g. MUC5AC, CK7 and CLDN18) in both CrD-SBAs and associated non-neoplastic mucosa [33] may suggest an early lineage commitment towards gastric differentiation in inflamed CrD mucosa, possibly promoting dysplastic and, subsequently, neoplastic evolution. Indeed, while in the stomach the loss of CLDN18 protein was reported to promote inflammation and tumorigenesis, as described in knockout models [43], de novo expression of CLDN18 in small bowel and colon-rectum of IBD patients seemed to be part of the inflammation-metaplasia-dysplasia-cancer process [44, 45]. In this regard, up to now, very scarce knowledge is available, and further investigations are required.

As expected, in line with another study on gastric neoplasms [27], CLDN18 was negatively associated with CDX2 expression in SBAs also. This finding suggests, in the light of the low expression of CLDN18 expression in CDX2-positive gastric cancers [46], a strong influence of the activation of highly specific intestinal transcription factor CDX2 in the differentiation of neoplastic cells towards an intestinal phenotype. The higher expression of intestinal differentiation markers described in CoD-SBAs in comparison with CrD-SBAs [8] may contribute to explain the low prevalence of CLDN18 positivity in CoD-SBAs.

Furthermore, we noted a negative correlation between CLDN18 and nuclear translocation of β-catenin. Nevertheless, CLDN18 has been described to have a two-faced behaviour towards β-catenin, both inhibiting and enhancing its expression [13]; thus, further molecular studies are needed to shed light on this correlation. Finally, we showed a non-significant trend towards a worse cancer-specific survival in CLDN18-positive SBAs, as reported in CRC [27], even though very discordant data are reported in the Literature for other gastrointestinal cancers [25 26 28 37 42 47 48].

Our study has some limitations, including its retrospective nature, as well as the limited number of cases, without a significant percentage of stage IV disease. However, due to the rarity of primary SBAs and the limited targeted therapies against advanced SBAs, information derived from this investigation might be considered in future clinical trials with CLDN18-directed drugs enrolling SBA patients.

In conclusion, we found that 28% and 6% of SBAs, mainly sporadic or CrD-related, expressed CLDN18 in ≥1% (of any intensity) and ≥75% (score 2+/3+) of tumour cells, respectively, suggesting that CLDN18 may be a potential therapeutic target even in a fraction of SBAs, and that MUC5AC+/CK7+ SBAs harbour the highest probability to exhibit immunoreactivity for CLDN18.

## Data Availability

The datasets generated during and/or analysed during the current study are available from the corresponding author on reasonable request.
